# Eco-Friendly Green Synthesis of Rubropunctatin Functionalized Silver Nanoparticles and Evaluation of Antibacterial Activity

**DOI:** 10.3390/nano12224052

**Published:** 2022-11-17

**Authors:** Guibin Lin, Chenhui Zhao, Wenqiang Liao, Jianmin Yang, Yunquan Zheng

**Affiliations:** 1 College of Chemistry, Fuzhou University, No. 2 Xueyuan Road, Fuzhou 350108, China; 2 College of Biological Science and Engineering, Fuzhou University, No. 2 Xueyuan Road, Fuzhou 350108, China; 3 Fujian Key Laboratory of Medical Instrument and Pharmaceutical Technology, Fuzhou University, No. 2 Xueyuan Road, Fuzhou 350108, China

**Keywords:** green synthesis, silver nanoparticles, rubropunctatin, antibacterial

## Abstract

In order to solve the problems of rubropunctatin insoluble in water and its low bioavailability, and explore the synthesis method of green silver nanoparticles, rubropunctatin was used as reducing agent and blocking agent, rubropunctatin-functionalized silver nanoparticles (R-AgNPs) were successfully synthesized. The distinctive absorption peak at 410 nm confirmed the formation of R-AgNPs. Zeta potential measurement showed excellent stability of R-AgNPs with negative values of −29.81 ± 0.37 mV. The results of TEM and XRD showed that the prepared R-AgNPs were round, well dispersed and crystallized with average particle size of 13.54 ± 0.42 nm. FT-IR and XPS studies show that functional groups are involved in R-AgNPs synthesis. The antibacterial activity of R-AgNPs was compared with commercial silver nanoparticles (AgNPs) by microdilution method. The results showed that R-AgNPs (MIC 7.81 μg/mL) has stronger antibacterial activity than commercial AgNPs. The bacteria morphology was observed by the live and dead (SYTO 9/PI) staining assay and SEM showed that the antibacterial effect of R-AgNPs was caused by the destruction of the bacterial cell membrane. Cytotoxicity of rubropunctatin-functionalized silver nanoparticles and commercial silver nanoparticles on mouse fibroblast 3T3 cells was assessed by CCK-8 assay. The results showed that the toxicity of rubropunctatin-functionalized silver nanoparticles to 3T3 cells was lower than that of commercial silver nanoparticles. In summary, synthesis of silver nanoparticles using rubropunctatin is a green synthesis method, and R-AgNPs is a potential antibacterial agent.

## 1. Introduction

Since ancient times, human beings have been struggling with infectious diseases caused by bacteria, fungi, viruses and other pathogens. These microorganisms have the characteristics of small size, rapid reproduction, strong adaptation, variable frequency, wide distribution and numerous varieties [1]. Bacterial infections are referred to as the most numerous of all human diseases [2]. The discovery of antibiotics is one of the most important achievements of modern medicine [3,4]. However, the abuse of antibiotics in clinical and non-clinical settings has led to the emergence of many drug-resistant bacteria called superbugs, such as methicillin-resistant *S. aureus* bacteria (MRSA) and Vancomycin *S. aureus* (VISA) [5,6,7]. As a result, bacteria remain one of the biggest threats to human health. Researchers have developed a variety of antimicrobial agents to inhibit bacterial proliferation and even kill bacteria. Up to now, the commonly used antibacterial agents are mainly divided into natural antibacterial agents, inorganic antibacterial agents and organic antibacterial agents [8]. However, with the increasing variety and quantity of antibacterial products, the resistance of microorganisms increases, and causes serious environmental problems [9]. Therefore, it is urgent to develop effective antibacterial materials as substitutes for these drugs.

With the continuous progress of science and technology, the rapid development of nano-science and technology has opened up a new way for antibacterial drugs. Silver nanoparticles (AgNPs), the most common silver-containing nano-materials are processed to nano-scale silver particles [10]. As a kind of high-efficiency broad-spectrum antibacterial materials, silver nanoparticles have been broadly used in daily life, including antibacterial fabrics, water purification, antibacterial food packaging and antibacterial coatings [11,12,13,14,15,16]. However, the traditional method of AgNPs synthesis is to use organic phase synthesis method to obtain monodisperse nanoparticles, but organic solvents are generally toxic which pose a great threat to the environment and human body [17]. Traditional AgNPs synthesis methods are not suitable for biomedical applications, and thus it is necessary to transfer nanoparticles from organic phase to aqueous phase, but this process often requires the involvement of phase transfer agents and cumbersome surface modification process [18]. It is now necessary to find a simple, green and safe way to synthesize silver nanoparticles without creating toxic waste in the process [19]. Therefore, researchers devote to the cost-effectiveness, eco-friendliness and wide applications of hydrothermal synthesis of nanoparticles of natural active substances.

Monascus pigment is a secondary metabolite produced during the growth of Monascus [20]. It is widely used as a natural food pigment and has good redox activity, anti-corrosion and anti-cancer biological activity [21,22,23]. Rubropunctatin is a kind of orange pigment in monascus pigment with a range of biological activities and pharmacological activities, such as antibacterial, anti-Alzheimer’s disease, anti-inflammatory, antioxidant and anti-tumor [24,25,26,27,28]. The use of natural food pigments with antibacterial activity as reductant and blocking agent is helpful to prepare nanoparticles with enhanced antibacterial activity [29]. However, rubropunctatin is not widely used in clinic for its poor water solubility and low availability [30]. In order to solve these problems, researchers have designed many schemes and carried out a large number of experimental studies, which mainly including change the dosage form, modify the structure of rubropunctatin and look for the suitable drug carrier [24,30,31]. Therefore, how to better load and transport rubropunctatin and how to promote the pharmacological activity of rubropunctatin have arose great interests of the scholars.

For the purpose of synthesizing AgNPs in a green way, rubropunctatin-functionalized silver nanomaterials (R-AgNPs) with high antibacterial activity were studied based on the above analysis. Notably, it is necessary to further test the antimicrobial properties of R-AgNPs to provide green synthetic alternatives. The cytotoxicity of R-AgNPs and AgNPs was determined by CCK-8 kit. In addition, the hydrothermal synthesis of R-AgNPs is a green and safe method with potential applications.

## 2. Materials and Methods

### 2.1. Materials

The rubropunctatin was purified in our laboratory (purity ≥ 99%) [21]. Silver nitrate (AgNO_3_) and gentamicin were purchased from Sigma-Aldrich (St. Louis, MO, USA). AgNPs (60 nm, purity ≥ 99.5%) and gentamicin (purity ≥ 99%) were purchased from Macklin (Shanghai, China). *Escherichia coli* (*E. coli*) was provided by the Microbiology Laboratory, College of Biological Science and Engineering, Fuzhou University, Fuzhou, China. Staphylococcus aureus (*S. aureus*) was obtained from the BNCC and stored in our microbiology laboratory. Sterile LB medium was prepared in our laboratory. SYTO9/PI staining kits and CCK-8 kit were purchased from KGI Biotechnology Development Co., Ltd. (Nanjing, China). DMEM medium, trypsin and penicillin–streptomycin solution were obtained from Gibco BRL (Gaithersburg, MD, USA). Fetal bovine serum (FBS) was obtained from Invitrogen GmbH (Karlsruhe, Germany). PBS (pH 7.2) was obtained from Shanghai Yuanpei Biotechnology Co., Ltd. (Shanghai, China). Mouse fibroblast 3T3 cells was purchased from Cell Resource Center of Shanghai Biological Sciences Institute (Chinese Academy of Sciences, Shanghai, China). All other reagents (analytical grade) were purchased from Sinopharm Chemical Reagent Co., Ltd. (Shanghai, China) and used as received without additional purification.

### 2.2. R-AgNP Synthesis

Rubropunctatin was used as a reducing agent, and silver nitrate (AgNO_3_) was used as a precursor to synthesize AgNPs. Rubropunctatin (0.625 mg) was dissolved in 30 mL ethanol (purity ≥ 95%) and completely dissolved. A total of 15 mL of 1 × 10^−3^ mM aqueous solution of AgNO_3_ was mixed with 3 mL of the rubropunctatin. The mixture was kept at 80 ℃ oil bath for 60 min, stopped heating and then cooled the solution to room temperature. The color of solution became yellowish brown following reduction of silver ions. The products were centrifugated at 12,000 rpm for 20 min and precipitation was washed with deionized water three times. Finally, the silver product, named R-AgNPs, was obtained.

### 2.3. R-AgNP Characterization

The morphology, particle size, element and other characteristics of rubropunctatin functionalized silver nanoparticles were characterized by UV-Vis, Zeta, TEM, XRD, FT-IR and XPS.

The aqueous solution of rubropunctatin functionalized silver nanoparticles were analyzed by UV-Vis spectrophotometer (Model UV-1800, Suzhou, China). An amount of 3 mL of AgNO_3_, rubropunctatin-AgNO_3_ and rubropunctatin functionalized silver nanoparticles were obtained, respectively. The successful synthesis of rubropunctatin functionalized silver nanoparticles was judged by comparing their plasma resonance absorption spectra in the range of 300–800 nm.

The particle size distribution and zeta potential of rubropunctatin functionalized silver nanoparticles were measured with a Zetasizer Nano series (Model, Zetasizer Nano ZS, USA) analyzer. A suitable amount of sample solution was mixed with purified water at a volume ratio of 1:50. After 10 min of ultrasound, the measurement was performed.

The average particle size, surface morphology and dispersion of rubropunctatin functionalized silver nanoparticles were measured by TEM (Model, Tecnai G2 F30, USA). After ultrasonic treatment for 30–60 min, 1–3 suspension droplets of rubropunctatin functionalized silver nanoparticles were placed on a copper mesh and then dried to observe the microstructure of rubropunctatin functionalized silver nanoparticles.

The rubropunctatin functionalized silver nanoparticles solution was drop-casted onto a silicon wafer, and the XRD pattern was obtained from Bruker D8 advance powder X-ray Cu-ka radiation diffractometer. The test conditions are as follows: the X-ray source (λ = 1.540598) is a Cu-Ka line, the tube voltage is 40 kV, the tube current is 20–30 mA, the scanning angle is 30–80° and the scanning speed is 5°/min.

Identification of functional groups in presents of rubropunctatin and rubropunctatin functionalized silver nanoparticles was carried by FT-IR (Model, Nicolet Nexus 870, USA), which scanning test range is 500–4000 cm^2^. At first, 200 mg of spectral pure potassium bromide was ground with 2.0 mg of rubropunctatin and rubropunctatin functionalized silver nanoparticles, respectively, and then put into a tablet press (pressure 10 MPa, Time 3 min) for sample preparation, the test can be performed by putting the tablet into the instrument after pressing.

The elemental composition of the synthesized was detected by XPS (Model, VG ESCALAB250, USA). An amount of 20 mg of rubropunctatin functionalized silver nanoparticles was measured by X-ray photon beam. Test parameters: Al Target (ka = 1486.6 eV), background subtracted by Sherry function, Gauss–Lorentz function fitting curve, C1s binding energy 284.6 eV as reference.

### 2.4. Antibacterial Activity Assay

Microdilution method was carried out to assay the antibacterial activity of rubropunctatin functionalized silver nanoparticles against two bacterial pathogens, namely, *E. coli* and *S. aureus*. *E. coli* and *S. aureus* were inoculated into LB liquid medium (20 mL) and cultured at 220 rpm at 37 °C for 8 h, then the absorbance values at 600 nm were measured. Dilute the bacterial solution to 1 × 10^6^ CFU/mL with sterile PBS (pH = 7.2).

Minimum inhibitory concentration (MIC) and minimum bactericidal concentration (MBC) of R-AgNPs. Using the 96-well plate to measure the value of MIC and MBC. Each well contained 100 μL Mixed medium, 5 μL of 10^6^ CFU/mL bacterial suspension. An amount of 100 μL of R-AgNPs, rubropunctatin, AgNPs and gentamicin at different concentrations (4000, 2000, 1000, 500, 250, 125, 62.5, 31.25, 15.625, 7.8125, 3.906 and 1.953 μg/mL) were incubated overnight at 37 °C. The negative control wells contained only 100 μL LB liquid medium and 5 μL bacterial suspension. MIC was the lowest concentration that visibly inhibited bacterial growth. To determine the MBC, 10 μL mixed medium with no bacterial growth on the surface was taken from the transparent well and cultured in solid medium at 37 °C for 24 h. The minimum concentration at which bacteria cannot grow corresponds to the MBC.

The bacterial survival rates of R-AgNPs, AgNPs and rubropunctatin were evaluated at different concentrations. An amount of 10 μL bacterial suspension (1 × 10^6^ CFU/mL) was added to R-AgNPs, AgNPs and rubropunctatin solution at 2MIC and shaken in the shaker at 120 rpm at 37 °C for 24 h. Then OD600 was measured by enzyme-linked immunosorbent (Model, Multiskan FC, Shanghai, China) and the survival rate of bacteria was calculated.

The bacterial growth curves were evaluated at different concentrations of R-AgNPs. 1 × 10^6^ CFU/mL bacterial suspension was divided into 5 equal components and a series of concentrations of R-AgNPs was added at 37 °C. The 96-well plate was cultured in 37 °C incubator. After shaking for 0, 3, 6, 9, 12 and 24 h, OD600 was measured by enzyme-linked immunosorbent (Model, Multiskan FC, Shanghai, China), and the growth curve of bacteria was drawn.

The viability of bacteria in R-AgNPs was evaluated quantitatively by the live and dead (SYTO 9/PI) staining assay. An amount of 1 × 10^6^ CFU/mL bacterial suspension was incubated with 2MIC of R-AgNPs for 4 h, without R-AgNPs as control, and centrifuged at 10,000 rpm for 3 min. Washed with PBS (pH 7.2) and distribute evenly with 1 mL bacterial suspension. After adding PI at the concentration of 50 μg/mL, the bacteria were incubated in darkness for 15 min, centrifuged and washed once, then mixed with SYTO 9 at the concentration of 1 μg/mL, incubated in darkness for 15 min, centrifuged and washed once, and was distributed evenly with PBS (pH = 7.2). An amount of 10 μL of bacterial suspension, prepared as above, was placed on the glass slide and then observe and recorded with the fluorescence microscope (Model, Nikon-50i, USA).

SEM was used to observe and evaluate the morphology of bacteria before and after administration. The suspension with concentration of 1 × 10^6^ CFU/mL was incubated with R-AgNPs for 2 h. Collection was centrifuged at 10,000 rpm for 3 min, washed and distributed evenly with PBS (pH = 7.2). Gradient dehydration was then performed by using 30%, 50%, 70% 80%, 90% and 100% ethanol. Finally, the samples were dried by freeze-drying method, and the morphology of bacteria was observed by SEM (Model, Verios G4, USA) after gold spraying.

### 2.5. Assessment of Cytotoxicity by CCK-8 Assay

The cytotoxicity of R-AgNPs and AgNPs at different concentrations was evaluated. The source of the mouse fibroblast 3T3 cells was embryonic fibroblasts from NIH Swiss mice. The CCK-8 assay was used to evaluate the cytotoxic effect of R-AgNPs and AgNPs on 3T3 cells with a 96-well plate. 3T3 cells were cultured in DMEM with 10% FBS, 1% penicillin–streptomycin medium and 1% gentamicin at 37 °C in a humidified atmosphere with 5% CO_2_ for 3 days. When 3T3 cell density reached 80%, they were digested by trypsin for 4 min and cell density was regulated to 104 cells/mL by DMEM. An amount of 100 μL cell suspension (1 × 10^4^ cells/mL) was seeded in 96-well culture plates and was cultured for 24 h, R-AgNPs and AgNPs were sterilized by 0.22 μm microporous filter membrane, and mixed with DMEM in a 1:1 ratio, the final mass concentrations of R-AgNPs and AgNPs were 20, 40, 60, 80 and 100 μg/mL, respectively. The mixture was cultured in a humidified atmosphere at 37 °C and 5% CO_2_ for 24 h, and the cell activity was detected based on the instructions of CCK-8 kit.

## 3. Results

### 3.1. Visible Observation and UV-Vis Spectroscopic Analysis

**Figure 1:**
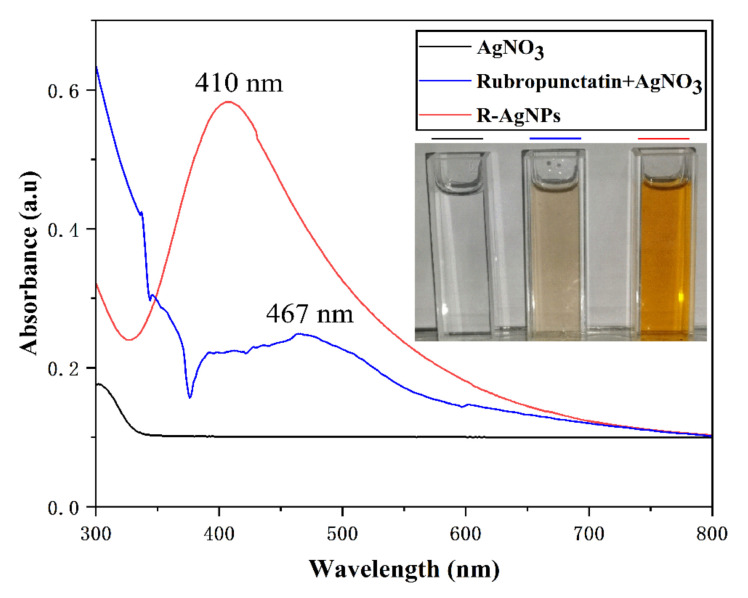
Image of the changing color of the reaction mixture before and after adding silver nitrate and UV–Vis spectrum of the R-AgNPs.

Visually, the silver nitrate is dispersed in rubropunctatin, resulting in an initial light orange color that gradually turns dark orange as the reaction progresses, as shown in Figure 1. The color change in the solution indicated that Ag^+^ was reduced to Ag^0^, which confirmed the formation of R-AgNPs [32]. The formation of R-AgNPs was further monitored and confirmed by UV-Vis spectroscopy. Due to the surface plasmon resonance (SPR) phenomenon of R-AgNPs, R-AgNPs exhibits a visible UV absorption peak at 410 nm which is consistent with the SPR absorption peak of AgNPs in the range of 400–420 nm [33]. On the other hand, no SPR peak was shown in the silver nitrate solution at 300–800 nm, whereas the characteristic peak of rubropunctatin appeared at 467 nm in the rubropunctatin and silver nitrate mixture. This further confirms the production of R-AgNPs.

### 3.2. Particle Size Analysis and Zeta Potential Study of R-AgNPs

**Figure 2:**
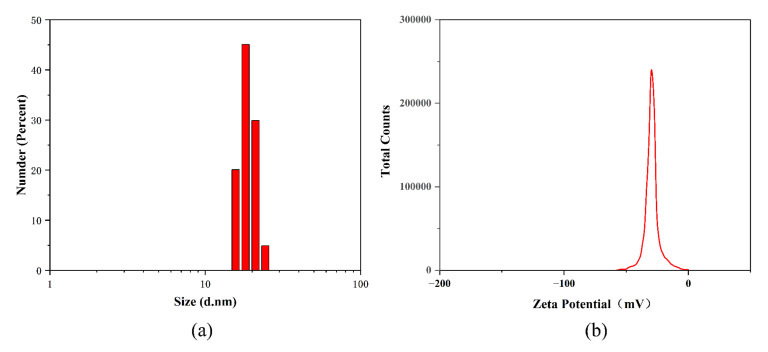
Particle size analysis (**a**) and Zeta potential (**b**) study of the R-AgNPs.

The results of granularity analysis of the synthesized R-AgNPs are shown in Figure 2a. The dynamic light scattering (DLS) particle size of R-AgNPs was 17.41 ± 3.61 nm. The polydispersity index (PDI) of R-AgNPs was 0.052 ± 0.038 that indicates greater dispersion.

Zeta potential is one of the determinants of nanoparticle stability. The smaller the nanoparticles, the higher the absolute Zeta potential (positive or negative). This means that the dispersion system of nanoparticles is more stable. The Zeta potential of the synthesized R-AgNPs was −9.2 ± 0.66 mV (Figure 2b), which indicated a high stability of the synthesized R-AgNPs.

### 3.3. TEM Analysis of R-AgNPs

**Figure 3:**
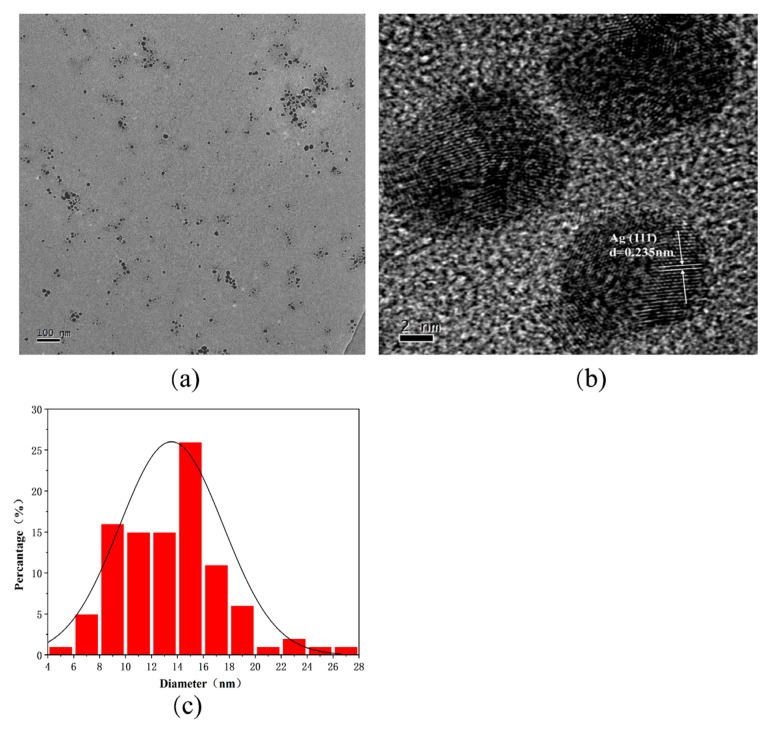
(**a**) TEM image of R-AgNPs, (**b**) HR-TEM images of the R-AgNPs, (**c**) histograms of silver nanoparticles in TEM.

The morphology and crystal structure of R-AgNPs were evaluated by TEM. The R-AgNPs showed a spherical structure with uniform morphology and good dispersion (results (Figure 3a). The average particle size of 100 nanoparticles was 13.54 ± 0.42 nm (Figure 3c). The results show that the synthesized R-AgNPs has good stability. The microstructure and crystallinity of R-AgNPs were observed by high-resolution transmission electron microscopy (HR-TEM). Spherical MPs–AgNPs show clear and uniform lattice fringes, which explained the MPs–AgNPs crystalline nature. The crystal plane spacing of R-AgNPs is 0.235 nm by HR-TEM, which indicates that the silver atoms are mainly located on the (111) peak (Figure 3b). It is confirmed that AgNPs have good crystallinity and mainly composed of silver.

### 3.4. XRD Analysis of R-AgNPs

**Figure 4:**
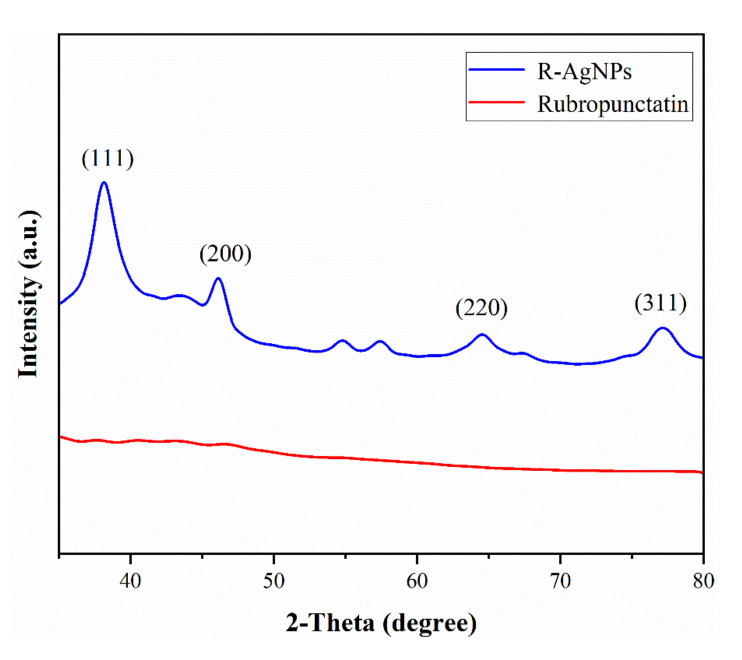
X-ray powder diffraction pattern of the R-AgNPs and rubropunctatin.

The X-ray-diffraction result (Figure 4) of rubropunctatin-reduced AgNPs shows that the R-AgNPs formed are crystalline. In the XRD diffraction pattern, the intense peaks at 38.24°, 46.47°, 64.61° and 77.42° was observed, which is corresponding to (111), (200), (220) and (311) Bragg’s reflections planes (ICSD No. 98-018-0878). The intensity of (111) diffraction peaks was much higher than other peaks, which indicated that R-AgNPs grew preferentially along (111) surface, which coincides with the results of HR-TEM. Low intensity diffraction peaks were observed at 54.21° and 58.36°, which were the bioactive residues produced by rubropunctatin during the reaction [32].

### 3.5. FT-IR Analysis of R-AgNPs

**Figure 5:**
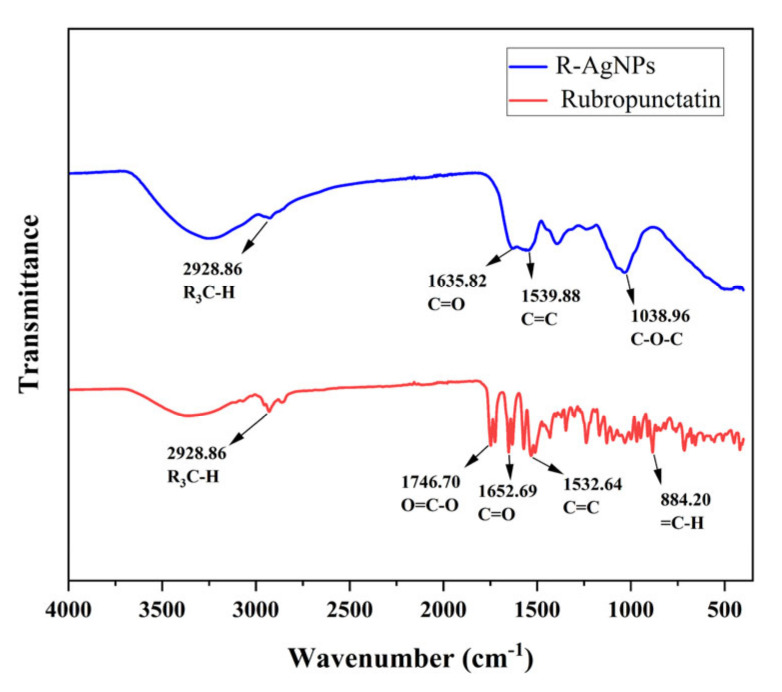
FT-IR spectra of R-AgNPs and rubropunctatin.

FT-IR spectra of the rubropunctatin and R-AgNPs were obtained in the 500–4000 cm^−1^ range in Figure 5. The FT-IR peak of Rubropunctatin show typical frequency bands at 1746.38 cm^−1^ (O=C-O), 1652.69 cm^−1^ (C=O) and 1532.64 cm^−1^ (C=C), which prove the existence of lactone ring. FT-IR spectra of R-AgNPs show peaks at 1635.82 cm^−1^ (C=O), 1539.88 cm^−1^ (C=C) and 1038.96 cm^−1^ (C-O-C). The lactone band of 1746.38 cm^−1^ (O=C-O) disappeared in the FT-IR spectra of R-AgNPs. This can be attributed to the lactone ring opening of rubropunctatin during the hydrothermal reaction and the reduction of silver nitrate to form an unsaturated carboxylic acid occurring at 1635.82 cm^−1^ (C=O). The peak value at 1038.96 cm^−1^ is just within the flexural vibration range of C-O-C group. FT-IR spectra show that the functional groups of rubropunctatin was involved in the synthesis of R-AgNPs. The opening of rubropunctatin lactone ring and the appearance of C-O-C are the main reasons for the synthesis of AgNPs. This corresponds to the presence of biological residues in XPS analysis.

### 3.6. XPS Analysis of R-AgNPs

**Figure 6:**
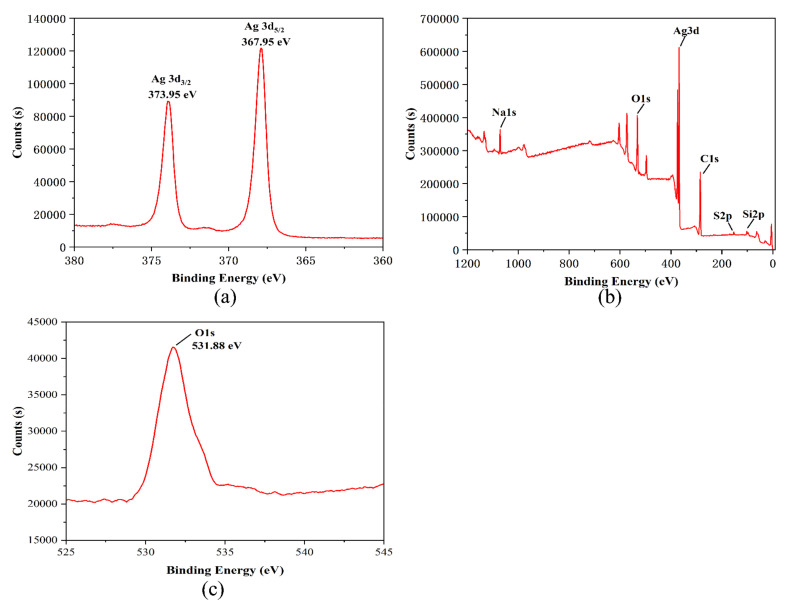
(**a**) XPS analysis of silver elements in R-AgNPs, (**b**) XPS analysis of all elements in R-AgNPs, (**c**) XPS analysis of oxygen in R-AgNPs.

The analysis of R-AgNPs by X-ray electron spectroscopy (XPS) is shown in Figure 6. As shown in Figure 6a, the binding energies of silver in R-AgNPs are 373.95 eV and 367.95 eV, and the distance between a pair of binding peaks is approximately 6.00 eV. This indicates that Ag^+^ is reduced to Ag^0^ by rubropunctatin [34]. Figure 6b shows that the chemical composition of the R-AgNPs surface is mainly composed of C, O and Ag elements. Figure 6c shows that the binding energy of oxygen in R-AgNPs is 513.88 eV. Table 1 shows that the contents of silver, carbon and oxygen in R-AgNPs are 9.44%, 57.74% and 22.22%, respectively. This indicates that the composition of R-AgNPs is biological residues of rubropunctatin and silver. This is consistent with previous studies [35].

### 3.7. Antimicrobial Activity

Table 2 presents the quantitative MIC and MBC of rubropunctatin functionalized silver nanoparticles (R-AgNPs). The MIC and MBC of commercial silver nanoparticles (AgNPs), rubropunctatin and gentamicin were also simultaneous measured and shown in Table 2. The MIC and MBC of rubropunctatin against for *E. coli* and *S. aureus* are relatively high, which revealed it has only weak antibacterial ability. The MIC and MBC of R-AgNPs for *E. coli* and *S. aureus* are 7.81 μg/mL and 15.62 μg/mL, respectively. The MIC and MBC of AgNPs for *E. coli* and *S. aureus* are 250 μg/mL and 500 μg/mL, respectively. These data show that rubropunctatin could inhibit bacteria far less than rubropunctatin functionalized silver nanoparticles prepared by using rubropunctatin as a reducing agent, and rubropunctatin functionalized silver nanoparticles had stronger antibacterial activity than commercial silver nanoparticles, which indicates the advantages of rubropunctatin functionalized silver nanoparticles compared to commercial silver nanoparticles in bacteriostasis.

**Figure 7:**
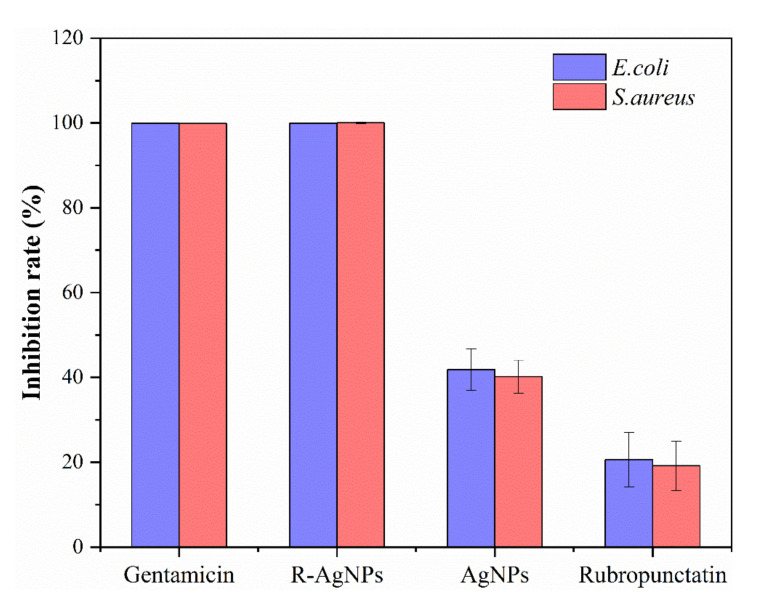
Inhibition ratio of R-AgNPs at 2MIC against *E. coli* and *S. aureus*. AgNPs, rubropunctatin and gentamicin were investigated for comparison.

The inhibition ratio of the R-AgNPs, and the control samples at 2MIC were further measured as shown in Figure 7. The inhibitory effect of R-AgNPs on *E. coli* and *S. aureus* was similar to that of the control gentamicin. This indicated that R-AgNPs had a high inhibitory rate against *E. coli* and *S. aureus* at 2MIC. R-AgNPs inhibited *E. coli* and *S. aureus* more than rubropunctatin and AgNPs. These results demonstrated that R-AgNPs have high inhibition ratio for *E. coli* and *S. aureus* at 2MIC which further confirm that the R-AgNPs have high antibacterial efficiency.

**Figure 8:**
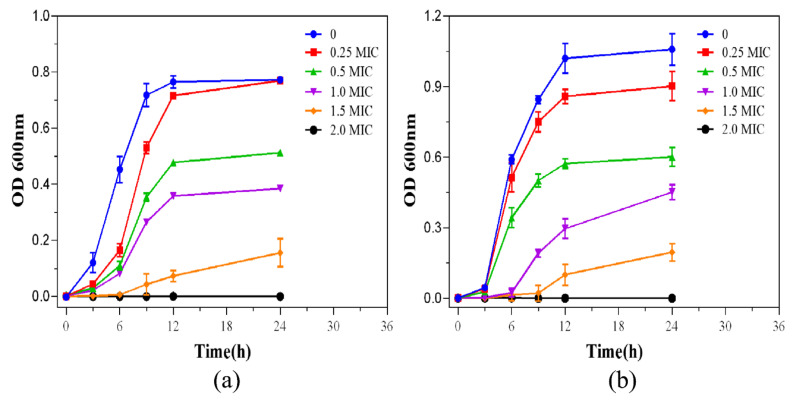
Growth inhibition curves of *E. coli* (**a**) and *S. aureus* (**b**) at different concentrations.

In order to study the inhibition of R-AgNPs on the growth of bacteria, the absorbance of bacteria at 600 nm was measured by enzyme-linked immunosorbent and the growth inhibition curves were made (Figure 8). It can be seen that with the increase in the concentration of R-AgNPs (0, 0.25 MIC, 0.5 MIC, 1 MIG, 2 MIC), the growth of bacteria becomes slower. The results showed that the addition of R-AgNPs to the bacteria inhibited the growth of *E. coli* and *S. aureus*.

**Figure 9:**
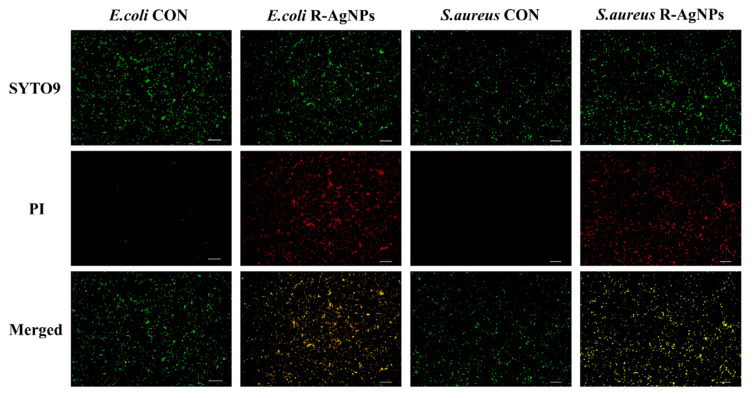
Fluorescence imaging of *E. coli* and *S. aureus* by R-AgNPs (2 MIC). Experimental conditions: 60× oil microscope observation. The scale is 100 μm.

To quantitatively assess bacterial survival in R-AgNPs, inverted fluorescence microscope analysis was performed by the dyes green fluorescent (SYTO 9) and propidium iodide (PI). It is well known that SYTO 9 is commonly used to stain cells and can even stain both living and dead cells, whereas PI can only stain dead or injured cells. As shown in Figure 9, only green fluorescence was observed in the control group without R-AgNPs treatment, indicating that the cell membranes of *E. coli* and *S. aureus* were intact. In contrast, a substantial overlap of green and red fluorescence was observed after treatment with R-AgNPs (2MIC), suggesting that R-AgNPs causes membrane disruption in *E. coli* and *S. aureus*. The results showed that R-AgNPs inhibited the growth of *E. coli* and *S. aureus* by destroying bacterial membranes.

**Figure 10:**
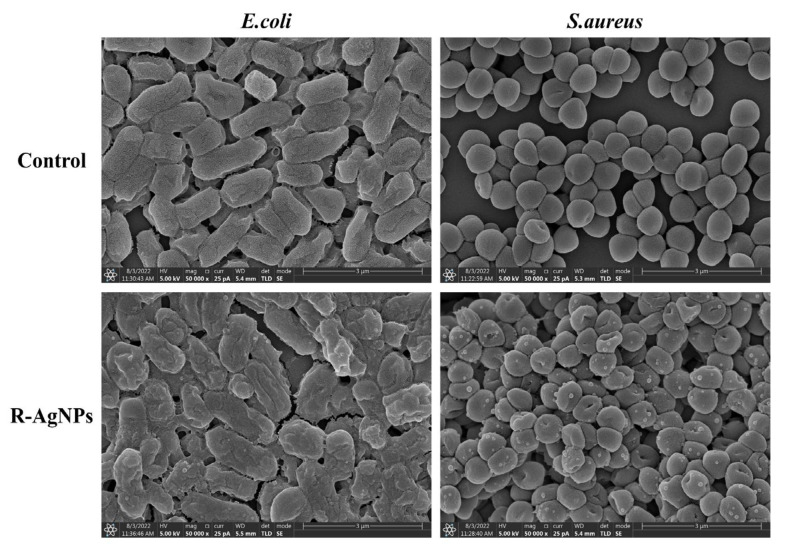
*E. coli* and *S. aureus* bacteria in R-AgNPs (2 MIC) detected by scanning electron microscopy. The scale is 3.00 μm.

To evaluate the antibacterial effect of R-AgNPs, SEM was used to monitor the morphological changes in *E. coli* and *S. aureus*. As shown in Figure 10, both R-AgNPs-treated *E. coli* and *S. aureus* had smooth surfaces, intact cell walls and good stereoscopic shape. In contrast, the surface of *E. coli* appeared obvious depression, the bacterial membrane was completely damaged and the inherent form of bacteria was seriously damaged after treatment with R-AgNPs (2MIC). *S. aureus* also showed change: the surface became rough and uneven with the partial inclusion material outflow and its membrane structure was less damaged than *E. coli*.

### 3.8. Assessment of Cytotoxicity by CCK-8 Assay

**Figure 11:**
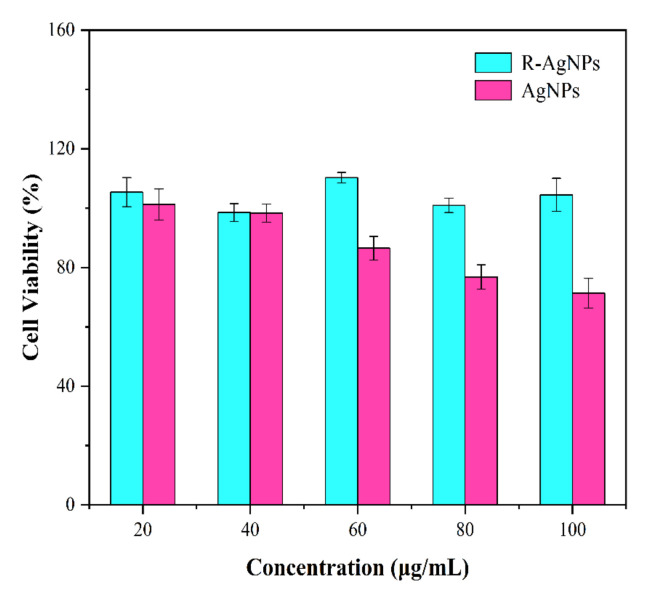
Cell viability of 3T3 cells in different concentrations of R-AgNPs and AgNPs.

CCK-8 assay was used to assess the cytotoxic effect of rubropunctatin functionalized silver nanoparticles (R-AgNPs) and commercial silver nanoparticles (AgNPs) by 3T3 cells. The cytotoxicity of rubropunctatin functionalized silver nanoparticles (R-AgNPs) and commercial silver nanoparticles (AgNPs) to 3T3 cells at different concentrations is shown in Figure 11. The cytotoxicity of rubropunctatin functionalized silver nanoparticles (R-AgNPs) to 3T3 cells was lower than that of commercial silver nanoparticles (AgNPs), which indicated that the activity of R-AgNPs was higher than that of AgNPs. At the low concentration of 40 μg/mL, neither R-AgNPs nor AgNPs showed cytotoxicity to 3T3 cells. At a concentration of 60 μg/mL, AgNPs began to show cytotoxicity to 3T3 cells while R-AgNPs still did not show cytotoxicity to 3T3 cells. These results illustrated that the cytotoxicity of rubropunctatin functionalized silver nanoparticles (R-AgNPs) to 3T3 cells was much lower than that of commercial silver nanoparticles (AgNPs).

## 4. Discussion

In the past decade, the reductive synthesis of green AgNPs by different natural active compounds has attracted the attention of researchers at home and abroad for its simplicity, environmental protection and safety [36]. Danni F. et al. used glycyrrhizin as a reducing and stabilizing agent to rapidly prepare low-toxic AgNPs with an average diameter of 35 nm (GL-AgNPs). GL-AgNPs have good antibacterial activity against *E. coli* (Gram-negative) and *S. aureus* (Gram-positive bacteria) [37]. Rui Z et al. rapidly synthesized MP-AgNPs with an average particle size of 18.10 ± 0.30 nm by monascus pigment (MP) under simulated sunlight, and demonstrated that the biosynthesized MP-AgNPs showed excellent inhibitory effects against *E. coli*, *P. aeruginosa* and *S. aureus* [33]. Di Filippo et al. synthesized bioactive AgNPs by using snail mucus extracted from spiral snail as a bio-reducing agent and bio-stabilizer. It was also confirmed that the bioactive AgNPs had strong antibacterial activity against *E. coli* (ATCC 25922) and *S. aureus* (ATCC 25923) [38]. In this study, rubropunctatin was used as blocking agent and reducing agent to prepare the R-AgNPs which showed good dispersion, uniform particle size and spherical character, with an average particle size of 17.41 ± 3.61 nm. The SPR peak of R-AgNPs at 410 nm can be obtained from UV-VIS spectrum. The Zeta potential of R-AgNPs can be obtained from the Zeta potential, indicating that the aqueous suspension of R-AgNPs is relatively stable. XRD analysis shows that R-AgNPs have crystal structure, mainly located on (111) crystal plane. FT-IR analysis showed that the main reason for the synthesis of R-AgNPs was the opening of lactone ring and the reduction of silver nitrate to form unsaturated carboxylic acid. XPS analysis showed that R-AgNPS was mainly composed of C, O and Ag, and Ag^+^ was reduced to Ag^0^ by rubropunctatin.

In this study, synthetic R-AgNPs showed better antibacterial activity than commercial AgNPs. As for the comparison between green synthetic AgNPs and commercial AgNPs, Barabadi H et al. used plant-synthesized (P-AgNPs) to show higher antimicrobial activity against *E. coli* (MIC 4 μg/mL), but P-AgNPs were highly toxic compared with commercial AgNPs [39]. The antibacterial activity and biocompatibility of R-AgNPs were both stronger than that of commercial AgNPs. Therefore, R-AgNPs will be identified as the preferred alternative to commercial AgNPs for antimicrobial applications. At present, the antibacterial mechanism of silver nanoparticles is not clear, but some researchers believe that biosynthetic AgNPs may affect the cell morphology of bacteria by destroying the cell walls of *E. coli* and *S. aureus* and penetrating the cell membrane, thereby reducing cell proliferation and ultimately leading to cell death [40]. In this study, R-AgNPs was shown to destroy the cell walls of *E. coli* and *S. aureus* by means of the live and dead (SYTO 9/PI) staining assay and SEM observation which is consistent with previous conclusions. In addition, the size and shape of AgNPs increased the release of Ag^+^ ions, and affecting their antibacterial activity as the surface area of AgNPs increased [41]. It is generally believed that AgNPs smaller than 25 nm that can directly penetrate the cell membrane, enter the bacterial cells and start cell lysis [42]. In this study, the average particle size of R-AgNPs was 17.41 ± 3.61 nm, while that of commercial AgNPs was 60 nm. The antibacterial effect of R-AgNPs was better than that of commercial AgNPs, which was consistent with the previous literature.

## 5. Conclusions

In conclusion of the present study, R-AgNPs were synthesized using rubropunctatin as a reductant and blocking agent. Most of the synthesized R-AgNPs were spherical, dispersed and crystallized with an average particle size of 13.54 ± 0.42 nm. The antibacterial activity of R-AgNPs was higher than that of AgNPs and rubropunctatin, which indicated that R-AgNPs could enhance the antibacterial activity of rubropunctatin and AgNPs that specified as a potential antibacterial agent. The cytotoxicity of rubropunctatin-functionalized silver nanoparticles (R-AgNPs) to 3T3 cells was lower than that of commercial silver nanoparticles (AgNPs), which make clear that the prepared rubropunctatin functionalized silver nanoparticles (R-AgNPs) hold characteristic of green and security. The rubropunctatin-functionalized silver nanoparticles (R-AgNPs) is synthesized using a kind of green method, which has the advantages of simplicity, high efficiency, economy and environmental protection, and solves the problems of rubropunctatin insoluble in water and low bioavailability. To sum up, the synthesis of silver nanoparticles from R-AgNPs is a better method than the traditional method. R-AgNPs has potential applications in future biomedical and food packaging fields. Although R-AgNPs have high efficiency in vitro, we suggest that antibacterial activity and toxicity in vivo are needed to be further explored before R-AgNPs can be applied.

## Figures and Tables

**Table 1:** The elements of R-AgNPs.

Element	Binding Energy (eV)	Atomic (%)
Ag3d	367.95	9.44
O1s	531.88	22.22
C1s	284.89	57.74
Na1s	1071.29	3.60
Si2p	101.82	6.36
S2p	168.14	0.64

**Table 2:** The MIC and MBC (in μg/mL) of the R-AgNPs, AgNPs, rubropunctatin and gentamicin against *E. coli* and *S. aureus*.

μg/mL	*E. coli*		*S. aureus*	
	MIC	MBC	MIC	MBC
R-AgNPs	7.81	15.62	7.81	15.62
AgNPs	250	500	250	500
Rubropunctatin	500	1000	1000	2000
Gentamicin	1.95	3.91	3.91	7.81

Abbreviations: MBC, minimum bactericidal concentration; MIC, minimum inhibitory concentration.

## Data Availability

Not applicable.
